# Sleep-time eating boosts exercise endurance

**DOI:** 10.1093/lifemeta/load029

**Published:** 2023-06-30

**Authors:** Jonas T Treebak

**Affiliations:** Novo Nordisk Foundation Center for Basic Metabolic Research, Faculty of Health and Medical Sciences, University of Copenhagen, 2200 Copenhagen, Denmark

## Abstract

**Figure:**
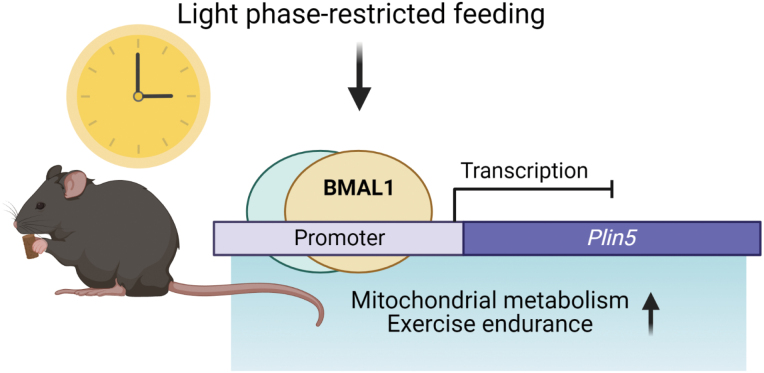
Light phase-restricted feeding in mice enhances exercise endurance in sedentary mice through a mechanism involving BMAL1-induced inhibition of *Plin5* expression (created with BioRender.com).

Light phase-restricted feeding in mice enhances exercise endurance in sedentary mice through a mechanism involving BMAL1-induced inhibition of *Plin5* expression (created with BioRender.com).

**Strategies for effective entrainment of the circadian clock by external cues are continuously being refined to circumvent dysfunctional cellular metabolism that may cause metabolic disease. The efficacy of daytime-restricted feeding was recently investigated in mice and shown to dramatically improve exercise capacity through a mechanism involving *Plin5*.**

Nutrients and physical activity are potent zeitgebers that act to entrain the autonomous clock present in all cells of our body. The molecular clocks consist of intricate transcription/translation feedback loops and oscillate in periods of about 24 h. Misalignment of zeitgebers and the circadian clock (i.e., chronodisruption) is thought to be a contributing factor to the dysfunctional cellular metabolism that can result in metabolic disorders [1]. Intermittent fasting is an umbrella term used to describe various feeding–fasting cycles. In recent years, there has been an increased interest in applying intermittent fasting in clinical settings as a strategy to prevent and treat metabolic disease [2]. Moreover, there are indications in the literature that both exercise performance and adaptations to exercise may depend on meal timing [3, 4]. However, the molecular underpinnings for how these adaptations occur as well as the putative involvement of the circadian clock in mediating the effects of intermittent fasting are incompletely known. This interaction among time-restricted feeding, the circadian clock, and exercise performance was investigated in mice in a recent study by Xin *et al.* published in *Nature Metabolism* [5].

Sedentary female and male mice were subjected to either ­daytime-restricted feeding (DRF; access to food in the light phase between Zeitgeber time 0 (ZT0) and ZT12), nighttime-restricted feeding (NRF; access to food in the dark phase between ZT12 and ZT0), or *ad libitum* (AL) feeding for 1–3 weeks, and exercise capacity was measured at ZT2, which falls in the phase when AL-fed mice exhibit high endurance [6]. While exercise performance was not affected by 1 week of DRF, 3 weeks of DRF resulted in a remarkable improvement in running endurance as measured by running time and distance by almost 100% in sedentary mice compared with NRF and AL feeding at ZT2. The effect persisted when tested at ZT14, and DRF increased running endurance even in the trained mice.

DRF was associated with major metabolic adaptations in skeletal muscle and increased the percentage of fast-twitch type 2A oxidative myofibers in the gastrocnemius muscle, along with higher oxidative bioenergetic activity and lower glycolytic activity. DRF also reduced liver weight and glycogen content, increased hepatic triglyceride level, and increased brown adipose tissue mass. DRF did not significantly affect the locomotor activity or food intake compared with the AL-fed group.

While skeletal muscle glycogen is not important for exercise capacity in mice [7], liver glycogen levels are known to play an important role in both exercise performance and substrate metabolism over the circadian cycle [6, 8]. Thus, the improved performance is somewhat surprising and indicates that repeated DRF rewires metabolism to efficiently utilize other substrates than glucose.

Interestingly, when mice underwent diet reversal from DRF to AL feeding, the difference in exercise endurance was eliminated after 3 weeks but not after 1 week. Diet reversal for 3 weeks also decreased the proportion of oxidative muscle fibers and increased the proportion of glycolytic fibers compared to DRF.

To investigate the role of the circadian clock in DRF-induced running endurance, the authors applied whole-body *Per1* and *Per2* double knockout mice as well as *Per2* null mice to show loss of the endurance-enhancing effect of DRF. On the contrary, the knockdown of *Per2* expression in skeletal muscle did not alter running endurance compared to control in either AL fed or DRF mice, suggesting the involvement of non-muscle organs in the DRF response. However, skeletal muscle-specific *Bmal1* knockout mice showed impaired running endurance under DRF, indicating the overall importance of the muscle clock in this response.

To explore potential molecular mechanisms underlying the regulation of exercise endurance by meal timing, a multi-omics approach was used. Transcriptomic analysis revealed that DRF reprogrammed the diurnal transcriptome in muscle, entraining *de novo* rhythmic genes. Enriched cycling pathways under DRF included regulation of mitochondria and lipid metabolic processes. Metabolomic and lipidomic analysis further supported the synchronization of mitochondrial lipid metabolism genes under DRF.

In subsequent analyses, the authors focused on four ­nucleus-encoded mitochondrial genes involved in lipid metabolism: *Plin5*, *Pdk4*, *Pdp2*, and *Hmgcs2*. These genes exhibited diurnal rhythms only under DRF but not NRF, and further investigation revealed that *Plin5* and *Pdk4* were the only genes that maintained rhythmicity in both male and female muscle tissues, while *Pdp2* and *Hmgcs2* lost rhythmicity in males. Disrupting the circadian clock through constant light abolished the rhythmicity of *Plin5* but did not affect *Pdk4*, *Pdp2*, and *Hmgcs2*. Additionally, whole-body knockout of *Per1* and *Per2* dampened the diurnal rhythms of *Plin5*, *Dbp*, and *Pdp2* without affecting *Pdk4* and *Hmgcs2* under DRF and constant dark conditions. Collectively, *Plin5*, which is a member of the perilipin family that coats intercellular lipid storage droplets and protects them from lipolytic degradation, was the only robustly rhythmic gene in males and females that required both a functional circadian clock and DRF to be rhythmic.

*Plin5* was also found to respond to exercise in human muscles. Exercise training resulted in decreased baseline expression of *Plin5*, and this response was observed in both young and older individuals, indicating the potential relevance of these findings to humans.

To determine whether the circadian clock and nutrient-sensing transcription factors directly regulate *Plin5* expression, a cistrome analysis was employed and showed enrichment of transcription factors such as PER2, BMAL1, CRY1, PPARA, and NR1D1 in the *Plin5* promoter regions. Moreover, BMAL1 was found to bind to the enhancer/promoter regions of the *Plin5* promoter, and ablation of the muscle clock through the knockout of *Bmal1* increased *Plin5* expression. This indicates that *Bmal1* acts to transcriptionally suppress *Plin5* expression under DRF, but how this relationship is regulated and whether transcriptional repressors are involved have yet to be elucidated.

Next, the specific role of skeletal muscle *Plin5* for DRF-enhanced exercise capacity was investigated using a knockdown mouse model where AAVs targeting *Plin5* transcripts by microRNA were injected in the tibialis anterior (TA) muscle. Remarkably, the knockdown of *Plin5* in the TA muscle resulted in increased running endurance during AL feeding, and this effect was not additive under DRF. *Plin5* knockdown in the TA muscle also led to a higher proportion of oxidative myofibers and lower glycolytic myofibers in the muscle of AL-fed mice. Using an alternative approach, where microRNA against *Plin5* was delivered systemically to target striated muscle tissues in general, this recapitulated the DRF-mediated increase in exercise capacity and was associated with higher oxidative and lower glycolytic capacity in the gastrocnemius muscle. Thus, *Plin5* knockdown phenocopied the effects of DRF.

Analyzing the transcriptomics profile of *Plin5* knockdown muscle, the researchers found that diurnal rhythms of rhythmic muscle genes and core circadian clock genes were not altered by *Plin5* knockdown. However, differential gene expression analysis identified 80 genes that were shared between *Plin5* knockdown and DRF. Pathway analysis revealed enrichment of lipid metabolic processes, ankyrin repeat-containing domain, and mitochondrion among these shared genes. Gene set enrichment analysis also showed activation of mitochondrial oxidative pathways in *Plin5* knockdown mice.

Metabolomic analysis of serum samples revealed distinct clustering between *Plin5* knockdown and wild-type mice. Acylcarnitine/fatty acids and amino acids were identified as the prominent features contributing to the clustering. The diurnal profiles of acylcarnitine/fatty acids and metabolites involved in the TCA cycle indicated a diurnal rhythm of circulating energy substrates in *Plin5* knockdown mice.

Overall, the study suggests that DRF entrains muscle *Plin5* rhythm in a clock-dependent manner and enhances running endurance through *Plin5*-mediated mechanisms that involve regulating lipid metabolism and mitochondrial bioenergetics. These findings are remarkable in the sense that substantial DRF-induced changes in exercise capacity and skeletal muscle metabolism occurred in sedentary mice. This implies that ­circadian-timed nutrient intake is a strong zeitgeber and an important mediator of skeletal muscle performance. While knockdown of *Plin5* in skeletal muscle phenocopied many aspects of DRF, it is worth noting that DRF had major effects on both liver and brown adipose tissue, and the contribution of these organs and how they crosstalk to potentiate exercise capacity needs further investigation. Finally, although Ramadan fasting, which corresponds to the experimental model applied in this study, has shown the potential to increase exercise performance in healthy humans [9], future studies should interrogate whether this dietary regimen has health benefits in people with metabolic disease.
